# Comprehensive identification of arginine methylation in primary T cells reveals regulatory roles in cell signalling

**DOI:** 10.1038/ncomms7758

**Published:** 2015-04-07

**Authors:** Vincent Geoghegan, Ailan Guo, David Trudgian, Benjamin Thomas, Oreste Acuto

**Affiliations:** 1 Laboratory of T cell signalling, Sir William Dunn School of Pathology, University of Oxford, Oxford OX1 3RE, UK; 2 Cell Signaling Technology Inc., Trask Lane, Danvers, Massachusetts 01923, USA; 3 Central Proteomics Facility, Sir William Dunn School of Pathology, University of Oxford, Oxford OX1 3RE, UK

## Abstract

The impact of protein arginine methylation on the regulation of immune functions is virtually unknown. Here, we apply a novel method—isomethionine methyl-SILAC—coupled with antibody-mediated arginine-methylated peptide enrichment to identify methylated peptides in human T cells by mass spectrometry. This approach allowed the identification of 2,502 arginine methylation sites from 1,257 tissue-specific and housekeeping proteins. We find that components of T cell antigen receptor signal machinery and several key transcription factors that regulate T cell fate determination are methylated on arginine. Moreover, we demonstrate changes in arginine methylation stoichiometry during cellular stimulation in a subset of proteins critical to T cell differentiation. Our data suggest that protein arginine methyltransferases exert key regulatory roles in T cell activation and differentiation, opening a new field of investigation in T cell biology.

## Abstract (web summary)

Arginine methylation is an important regulatory post-translational modification. Here, the authors present a new SILAC-based method—iMethyl-SILAC—that allows unambiguous identification of arginine-methylated peptide pairs by mass spectrometry and apply it to greatly expand the known T-cell arginine methylome.

## Graphical abstract

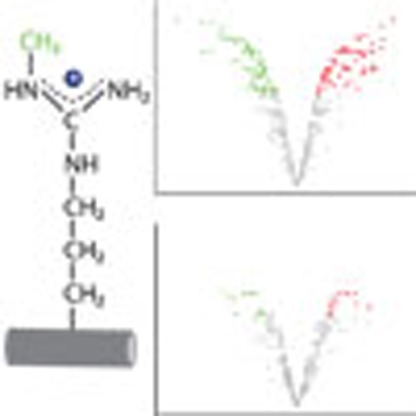

Post-translational modifications (PTMs) govern cellular homeostasis and responses to changes of internal and external conditions^1^. Thus, knowledge of the type and extent of PTMs in tissue proteomes should provide more exhaustive insights into physiological and pathophysiological mechanisms. Comprehensive mass spectrometry (MS)-based studies on highly reversible PTMs, such as protein phosphorylation and ubiquitination, have already revealed regulation of cellular signalling pathways correlating with physiological or pathological settings^2^,^3^. However, other PTMs have been more difficult to tackle at a global scale, such as protein arginine methylation, thought to be rather permanent^4^. In higher eukaryotes, protein arginine methylation can occur symmetrically or asymmetrically at the arginine side chain guanidino group and is mediated by at least nine different arginine methyltransferases (PRMTs)^4^. Methylation reduces the number (up to five) of arginine hydrogen bond donors weakening interactions in protein–protein and protein–nucleic acid complexes, potentially generating differential binding preferences^5^. However, arginine-aromatic, cation-pi bonds may be favoured by methylation as suggested for Tudor domain binding to symmetrically methylated arginine sites^4^,^6^,^7^. Mice deficient for PRMT1, PRMT4 or PRMT5 show embryonic or perinatal lethality, demonstrating the importance of this PTM^8^,^9^,^10^. Arginine methylation is an epigenetic histone modification^11^ and impacts on transcription and DNA-repair^12^ but the extent and potential plasticity of this PTM in cellular functions remains unclear. Initial MS-based proteomics investigations have been mired by inefficient enrichment for arginine-methylated peptides^13^,^14^,^15^. Moreover, confident identification of methylated sites in complex mixtures has been problematic due to the increased search space when matching fragmentation spectra^16^, as several amino acid substitutions are isobaric to methylation^14^. The elegant heavy methyl-SILAC labelling strategy by Ong *et al.*^14^ increased confidence in identification of methylated peptides, but it did not allow the discrimination between methylated and methionine-containing peptides.

Primary T cells from human blood are ideal tissues to investigate at proteomics scale the extent and impact of PTMs in normal and pathological cellular processes. Cell subsets can be readily purified in sufficient amounts with minimal manipulation and stimulated to differentiate *in vitro.* Thus, for example, resting naive or memory T cells can be induced by appropriate stimuli mimicking *in vivo* conditions, to turn into the effector cells that fend off microbial pathogens or tumours^17^ but also into T cells that initiate or control inflammatory responses^18^. The central role played by T cells in autoimmunity and inflammation^18^,^19^ make them an ideal target for monitoring alterations of PTM signatures in diseased individuals. T cells appear to be sensitive to perturbations of arginine methylation as T cell development is blocked in PRMT4-null embryos and earlier studies indicated that arginine methylation augments substantially during T cell activation^9^,^20^.

Here, we use isomethionine methyl-SILAC (iMethyl)-SILAC, an improved procedure to exclusively detect methylated peptides, different proteases and anti-mono-methylated arginine antibodies (Abs) recently described that effectively enrich for arginine-methylated peptides^21^. When applied to Jurkat T cells and TCR/CD28-stimulated primary T cells, this comprehensive approach allowed us to identify the largest number of arginine methylation sites and proteins known to date implicating PRMT action in most, if not all cell functions, including TCR-proximal signalling and cell fate programs. Furthermore, we demonstrated that arginine methylation stoichiometry changes during cell differentiation and show this to occur in mRNA splicing factors critical in T cell differentiation.

## Results

### Discovery of arginine methylation sites using iMethyl-SILAC

**Figure 1:**
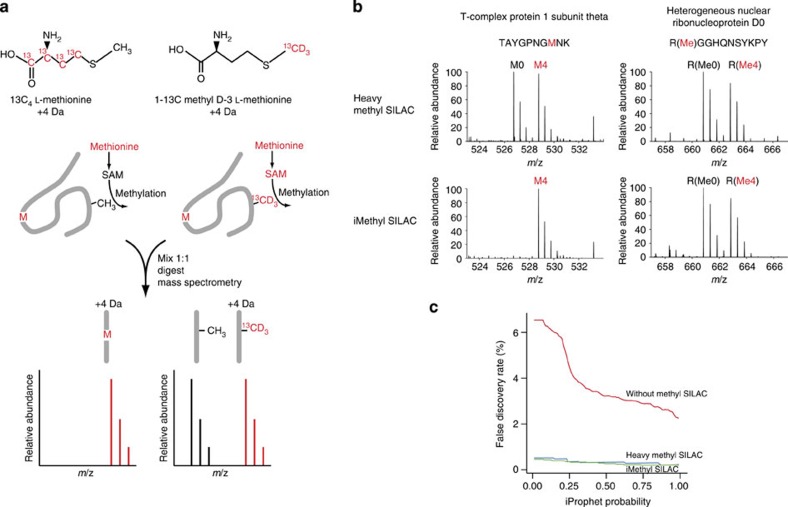
Isomethionine methyl-SILAC (iMethyl-SILAC) strategy. (**a**) Methionine is incorporated into proteins and also converted to the methyl group donor *S*-adenosyl methionine. Isotope-labelled methionines are nearly isobaric, but the distribution of label differs, therefore only methylated peptides will be observed as methyl-SILAC pairs. (**b**) Isomethionine methyl-SILAC labelling removes ambiguity arising from methionine-containing peptides. Methionine-containing peptides and methylated peptides are observed as indistinguishable methyl-SILAC pairs when cells are labelled by heavy methyl-SILAC (top). When cells are labelled by isomethionine methyl-SILAC, only methylated peptides give rise to methyl-SILAC pairs (bottom). (**c**) False discovery rate (FDR) among identified arginine-methylated peptides as a function of peptide identification confidence (iProphet probability). Primary T cells were labelled by heavy methyl-SILAC or iMethyl-SILAC. Without corroboration by methyl-SILAC pairs, the FDR rises rapidly to unacceptable levels as the minimum accepted iProphet probability is lowered (red line). By requiring a hit to be corroborated by a methyl-SILAC pair, the FDR is lowered in heavy methyl-SILAC-labelled samples (blue line) and further lowered in iMethyl-SILAC-labelled samples (green line).

In heavy methyl-SILAC, cells are labelled with L-Methionine or L-Methionine-^13^CD_3_. Presence of a 1:1 methyl-SILAC pair in the precursor scan corroborates the assignment of the fragmentation spectrum to a methylated peptide^14^. However, because the light or heavy methionine is incorporated into proteins, peptides containing methionine will also generate 1:1 methyl-SILAC pairs in precursor scans. To eliminate this ambiguity, we designed an improved labelling strategy, replacing L-Methionine with L-Methionine-^13^C_4_ (Fig. 1a). The two stable isotope-labelled methionines are nearly isobaric but differ in the distribution of the additional mass; we, therefore, termed this labelling strategy isomethionine methyl-SILAC (iMethyl-SILAC). The methyl groups transferred during protein methylation are still either light or heavy, but methionines incorporated during protein synthesis are nearly isobaric. As a result, methyl-SILAC pairs only arise from methylated peptides. To demonstrate the specificity and efficacy of iMethyl-SILAC, Jurkat T cells were labelled according to the heavy methyl-SILAC and iMethyl-SILAC strategies. Arginine-methylated peptides were immuno-affinity purified using mAbs D5A12 or MeR4100 raised against arginine mono-methylated sites in the context of various sequence contexts^21^ followed by LC-MS/MS (liquid chromatography-tandem mass spectrometry) analysis. When used in HeLa cells^22^ or HCT116 (ref. 21) cell extracts, these Abs were recently shown to effectively enrich for methylated peptides, which resulted in the identification of 1,000–1,700 arginine methylation sites. As expected, heavy methyl-SILAC labelling produced methyl-SILAC pairs from methionine-containing peptides and methylated peptides that were indistinguishable (Fig. 1b, top). In contrast, iMethyl-SILAC labelling meant that only methylated peptides occurred as methyl-SILAC pairs, providing unambiguous corroboration (Fig. 1b, bottom) and contributed to reducing the false discovery rate (FDR, Fig. 1c).

**Figure 2:**
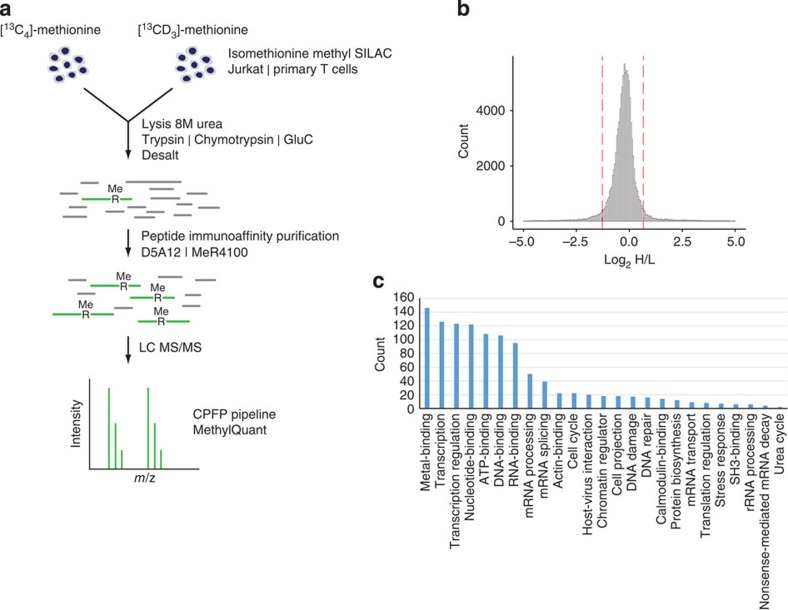
Comprehensive analysis of arginine methylation in T cells using iMethyl-SILAC for high confidence identification. (**a**) Experimental strategy. Isomethionine methyl-SILAC-labelled Jurkat or primary T lymphocytes are combined 1:1 and lysed. Proteins are digested with trypsin, chymotrypsin or GluC. Peptides are desalted and methylated peptides are enriched by immuno-affinity purification using D5A12 or MeR4100 antibodies. Putative methylated peptides are identified by mass spectrometry and matched to methyl-SILAC pairs. (**b**) All identified methyl-SILAC pairs. MethylQuant was used to quantify the methyl-SILAC pairs for each identified arginine-methylated peptide. Methyl-SILAC pairs with Heavy/Light (H/L) ratios between the 5th and 95th percentiles (dashed lines) were accepted. (**c**) Significantly overrepresented biological processes and molecular function involving identified arginine-methylated proteins.

We then performed a comprehensive analysis of arginine methylation using Jurkat T cells and peripheral blood lymphocyte-derived T cells from normal human donors that were activated with anti-CD3 and -CD28 Abs to allow iMethyl-SILAC labelling. Total cell extracts from Jurkat and primary T cells were digested with either one of three different proteases (Fig. 2a) and protein digests subject to immuno-affinity enrichment of methylated peptides using D5A12 or MeR4100 mAbs. LC-MS/MS detected a total of 82,338 methyl-SILAC pairs matched to arginine-methylated peptides, at an FDR of 0.93% (Fig. 2b). At the minimum accepted identification threshold (iProphet probability 0.25), iMethyl-SILAC led to a sixfold reduction in FDR when compared with label-free identification of methylation sites (Fig. 1c). When comparing iMethyl-SILAC and methyl-SILAC labelling, the FDR was further reduced, although to a lesser extent (Fig. 1c). Most of the methylated peptides were sequenced multiple times, which corresponded to 2,031 and 1,411 unique arginine methylation sites identified in Jurkat T cells and in primary T cells, respectively (Supplementary Fig. 1a and see below). Since an overlap of 940 methylation sites between Jurkat T cells and primary T cells was found (Supplementary Fig. 1a), together the two cellular sources gave a total number of 2,502 unique arginine methylation sites (Supplementary Data 1) derived from 1,257 unique proteins (Supplementary Data 2). This is the highest number of arginine methylation sites and proteins identified to date. Enrichment with the MeR4100 and D5A12 antibodies led to the identification of 1,939 and 1,421 unique sites, respectively (Supplementary Fig. 1b). On average, 11 or 6.7% of immuno-affinity purified peptides from primary T cells or Jurkat T cells, respectively, were arginine methylated (Supplementary Fig. 2), an approximate 560-fold enrichment as calculated from the 0.012% of arginine-methylated peptides present in Jurkat T cell lysate (Supplementary Fig. 2). Digestions with trypsin, chymotrypsin and GluC led to the identification of 2,147, 497 and 274 sites, respectively, with the latter two allowing detection of 355 additional sites not found using trypsin (Supplementary Fig. 1c). Reflecting the Abs specificity, the majority of methylation sites were identified as MMA (Supplementary Fig. 1d), which is considered to be an intermediate methylation state towards di-methylation^4^,^15^.

Using pLOGO^23^, we confirmed that arginine-methylated sites commonly occur in glycine-rich sequences (Supplementary Fig. 3). However, prolines were also overrepresented in close proximity to methylation sites (Proline Rich Arginine Methylation—PRAM—motifs)^15^, particularly at positions −1 and +4 relative to the methylated arginine. Proline at position +4 (*n*=380) was associated with a high occurrence of methionine and phenylalanine at position +1. The RXR methylation motif^24^ was observed as a significant overrepresentation of arginine at +2. Tyrosine at −4 was significantly overrepresented (*n*=88) and was associated with another tyrosine at +3. These findings should improve arginine methylation site predictions, although a sizable portion (*n*=675, 27%) of methylated sites substantially deviated from those motifs. Consensus outliers were previously noticed when methylated peptides were selected on the basis of hydrophilicity or negative charge content^15^. Moreover, some histone arginine methylation sites were not glycine-rich or PRAM motifs (Supplementary Data 1). These findings suggest that PRMT catalytic sites may be quite adaptable or that some PRMTs have propensity to methylate non-glycine-rich and non-PRAM sites and shows that the anti-monomethyl-arginine Abs used here are efficient at detecting a wide range of methylation sites.

### Arginine methylation is implicated in T cell fate decisions

In line with previous work, arginine-methylated proteins found in this study were implicated in regulating transcription (>200), post-transcription and translation (>230 in mRNA processing proteins, including eight proteins involved in RNA-mediated silencing) and chromatin re-modelling. Indeed, several components of histone modification machineries (for example, HAC, HDAC, lysine methyltransferases, JMJ) were found arginine methylated. As previously noted^15^, actin- and tubulin-mediated cytoskeleton re-modelling and intracellular protein transport, including nuclear pore components were also significantly enriched (170 proteins) among the identified methylated proteins (Fig. 2c). A novelty was the presence of 60 housekeeping enzymes (including several from mitochondria) involved in various metabolic pathways and ion channels. However, most surprising was to find arginine methylation sites in a relatively high number (∼100) in proteins forming signalling networks. We found that >40 protein and lipid kinases and phosphatases and 47 proteins that participate in ubiquitin conjugation in several cellular pathways were arginine methylated. Most of these sites and proteins were not detected in recent proteomics studies^21^,^22^. Remarkably, we found that adaptors/scaffolds and enzymes dedicated to TCR-proximal signalling (for example, LCP2/SLP76, PLCγ1, DOK1, GADS^25^), as well as several other well-known enzymes that regulate cellular signals leading to proliferation and/or survival (for example, SHIP1, SHP2, INPP5D, PIK3R1, PI3K, RasGRP2 and SOS2) were arginine methylated. This is the first time that receptor-proximal signalling components are clearly documented to be PRMT substrates^26^, opening new perspectives towards understanding signal tuning and diversification.

Of special interest was that arginine methylation was found in various general signalling pathways, such as Notch, Wnt and NF-kB, and in master transcription factors governing T-cell fate decision and maintenance^27^,^28^,^29^, as well as cytokine expression that contributes to driving T cell fates^28^ (for example, TCF3 and TCF12, LEF1, BCL11b, Notch1, Foxp3, Ikaros, Pax5, TBX21, Eomesodermin, RUNX1 and RUNX3, IRF4, NFAT1, cRel, RelB and see Supplementary Data 3). All these factors often interact directly or indirectly with each other and have crossover roles in other hematopoietic lineages and T cell subset establishment^27^,^29^. E-proteins play a critical role in lymphopoiesis, and TCF3 and TCF12 are required for B and T cell development but also for antigen-induced differentiation^27^,^28^. TBX21 is crucial for the development of CD4 T helper 1 (Th1) cells, promoting expression of interferon-γ (IFN-γ)^28^, the hallmark Th1 inflammatory cytokine. Eomesodermin, a paralogue of TBX21, is induced upon stimulation of naive CD8 T cells, and together with RUNX3 and TBX21 lead to expression of IFN-γ, perforin and granzyme B^30^. RUNX1 is expressed in Th2 cells and helps promote Th17 cell differentiation by up-regulating expression of RORγt, the Th17-signature transcription factor^18^. FoxP3, the master transcription factor required for regulatory T cell (Tregs) development so that these cells can limit autoimmunity and inflammation, cooperates with NFAT1, cRel and RUNX1 (ref. 18). Moreover, NOTCH1, Ikaros and Pax5 determine T and B cell fate at an early developmental stage but also during antigen stimulation^29^. Interestingly, we observed that none of the arginine methylation site of those factors fell within their respective DNA binding domains, suggesting that they may rather regulate domains that interact with various protein partners, which may be the case for RUNX1, RUNX3 and FOXP3 whose methylation sites were found at C-and N-regions moieties (Supplementary Data 3), respectively, previously mapped to interact with transcription partners^31^,^32^.

### Differential arginine methylation during T cell activation

**Figure 3:**
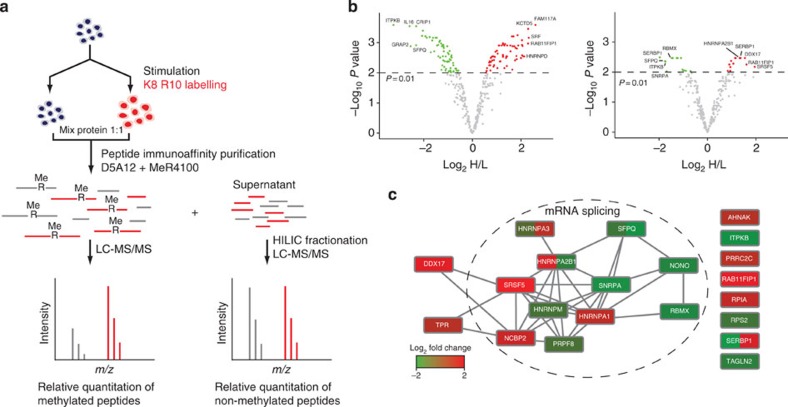
Quantifying changes in arginine methylation occupancy during primary T cell differentiation. (**a**) CD4+ T cells are isolated from human blood, a portion is collected directly (unstimulated). The remainder of the cells are stimulated with anti-CD3, anti-CD28 antibodies and IL-2. Cells proliferate and are stable isotope labelled for 7–9 days. Purified arginine-methylated peptides are identified and quantified. Non-methylated peptides found in the supernatant are fractionated and also identified and quantified. Quantification information is combined to calculate changes in arginine methylation occupancy. (**b**) Differential arginine methylation during T-cell differentiation. Thirty-seven per cent (134/365) of arginine-methylated peptides significantly increase (red) or decrease (green) in Heavy/Light (H/L) ratio during stimulation of T cells (left). After normalizing for changes in corresponding protein levels, >10% (27/282) of arginine-methylated peptides significantly increase (red) or decrease (green) in occupancy (right). (**c**) Interaction network of proteins showing changes in arginine methylation occupancy during T-cell stimulation. Proteins are coloured according to the change in arginine methylation occupancy, where a protein contains multiple sites, each is coloured individually.

We then set out to ask whether T cell activation induces changes in arginine methylation in general, as previously suggested^20^, and whether methylation sites undergo stoichiometry changes, as such sites would likely be important to T cell functions and differentiation (Fig. 3a). Primary CD4+ T cells from human blood were stimulated and labelled by conventional SILAC so that methylated and non-methylated peptides could be quantified. To maintain high confidence, methylated peptides were only accepted if they had also been identified in previous experiments by iMethyl-SILAC. We quantified 365 unique arginine-methylated peptides before and after stimulation of T cells, corresponding to 319 distinct arginine methylation sites in 202 proteins (Fig. 3b, left and Supplementary Data 4). A total of 134/365 (37%) peptides were altered in abundance as a result of stimulation. Because stimulation of T cells induces widespread changes in protein expression, non-methylated peptides remaining after IAP were fractionated by HILIC and analysed by MS to quantify changes in protein expression during stimulation. Changes in levels of methylated peptides were normalized to changes in protein expression to derive fold changes in occupancy of arginine methylation sites. This revealed that 27/282 (9.6%) arginine-methylated peptides did present altered methylation stoichiometry as a result of cellular stimulation (Fig. 3b, right and Supplementary Data 5), strongly suggesting arginine methylation-mediated dynamic regulation of a protein function, a notion that has not been convincingly explored before^4^. Although the number of site-specific changes in stoichiometry is likely to be an underestimate, the data indicate that a select group of proteins undergo changes in methylation occupancy during T cell differentiation. Among the methylation sites showing altered occupancy, a large proportion were found in proteins involved in mRNA splicing, some of them known to interact with each other in large complexes (Fig. 3c), and are candidates for regulating important T cell functions during differentiation^33^. In particular, SFPQ, which mediates TCR-signal induced alternative splicing of CD45 pre-mRNA, contains two carboxy (C)-terminal arginine methylation sites, which decrease in occupancy during T cell stimulation. These sites occur in the region known to interact with THRAP3 and are close to a phosphorylation site (Thr687), which also reduces in occupancy during T cell stimulation, allowing release of THRAP3 and association with CD45 pre-mRNA^34^. Changes in arginine methylation in the same region may modulate the splicing activity of SFPQ in response to TCR signals. PRMT4 was up-regulated during T cell stimulation and is known to methylate numerous splicing factors and influence patterns of alternative splicing^35^,^36^ (Supplementary Fig. 4).

## Discussion

Arginine methylation is still commonly regarded as a rare protein modification that has attracted limited attention in cell biology. The moderate interest for this PTM may be primarily due to the scarcity of PRMT substrates and sites known (SWISSProt reports so far less than a 100 arginine-methylated proteins); to its intrinsic stability, which is seen as incompatible with a role in regulating cellular pathways, as compared with intrinsically dynamic protein modifications (for example, phosphorylation, ubiquitination, acetylation); the belief that mostly generic cellular processes are regulated by PRMTs, as only a few pioneering functional studies exist so far on the subject^4^ and that detection of arginine methylation is technically very demanding and may carry unacceptable uncertainty for MS-based PTM identification. Our work demonstrates the widespread and dynamic occurrence of arginine methylation in primary cells and dispels earlier impressions, it can now be considered a PTM of major importance. Pan-Abs with specificity for arginine-methylated proteins/peptides largely independent of sequence context were recently generated^21^. They allowed considerable selection of methylated over non-methylated peptides (>500-fold purification, in this work), that together with iMethyl-SILAC labelling, development of software for methyl-SILAC pair matching (see Methods), use of multiple search algorithms and the use of different proteases made possible in our study a significant increase in the number of known PRMT substrates. Our work extends and improves upon very recent investigations that used the same enriching Abs but were mainly carried out in transformed cell lines (HeLa and HEK-293) and used label-free MS-based approaches. We found that identification of arginine-methylated peptides with a label-free approach carries more uncertainties (a higher FDR) in methylated peptide identification, requiring higher peptide identification probability thresholds that considerably reduces the number of sites identified. Although iMethyl-SILAC slightly improved FDR as compared with heavy methyl-SILAC previously described^14^, it should be still more effective for peptide mixtures where methylated peptides are less abundant. We therefore believe that minimizing the FDR to the lowest levels, as shown here to increase data set confidence, is a critical issue when detecting PTM sites at proteomics level, an incorrect assignment will mislead follow-up functional studies. Previous studies have provided convincing evidence that mono-methylated arginine is often a transition state toward di-methylation and it is likely that most methylations sites we found are transition states^4^.

The use of primary immune cells extended not only the PRMT substrates implicated in housekeeping functions and diverse cellular pathways but revealed that PRMTs control several T cell-specific functions. Thus, our and other recent studies^21^,^22^ open a new area of investigation to test in mouse models (for example, arginine mutations) and humans (searching for mutations-causing disease at methylation sites) the *in vivo* relevance of arginine methylation for cell fate decisions and hematopoietic lineage diseases. Master transcription factors govern differentiation often by associating in the same or different cell subsets with different sequence-specific transcription factors, co-activators and co-repressors building up transcriptional landscapes that favour a particular lineage while repressing expression of an alternative one. Such a behaviour requires structural flexibility likely conferred by a code composed of combinations of PTMs^1^, similar to histone modifications^37^, but also progressive stabilization towards more differentiated phenotypes that can be more permanently conferred by stable PTMs^5^. Progressive changes or *de novo* methylation in proteins during differentiation programs may represent a relatively stable record of earlier more dynamic PTMs activated by signalling events. Our study demonstrates that the augmentation of arginine-methylated proteins during a differentiation process previously observed^20^,^38^ is not only due to *de novo* protein expression of methylated proteins, but also to changes in stoichiometry. Thus arginine methylation can be dynamic, at least at the time scale of differentiation processes taking several hours or days as shown here. It will be interesting to investigate by our comprehensive approach alterations of these pathways that may lead to immune and inflammation pathologies. Together with recent similar investigations^21^,^22^, the present work raises arginine methylation to the rank of other more well-known PTMs, such as acetylation, phosphorylation and ubiquitination^2^,^3^,^39^.

## Methods

### Labelling of cells by SILAC

For SILAC labelling of cells, RPMI 1640 lacking L-methionine, L-arginine and L-lysine (Dundee cell products) was supplemented with 10% dialysed fetal bovine serum (GIBCO) and for methyl SILAC labelling either: (a) L-methionine, (b) L-methionine-^13^C_4_ (Sigma Isotec), (c) L-methionine-methyl-^13^CD_3_ (Sigma) all at 0.1 mM together with 0.29 mM L-arginine and 0.219 mM L-lysine. For conventional SILAC, 0.1 mM L-methionine, 0.29 mM L-arginine-^13^C_6_, ^15^N_4_ and 0.219 mM L-lysine-^13^C_6_, ^15^N_2_ (Cambridge Isotope Laboratories) were used. Jurkat cells were cultured at 37 °C for five to seven cell doublings in a humidified 5% CO_2_ atmosphere. Human primary CD4+ T lymphocytes were isolated from the blood of healthy donors by negative selection using a Dynal isolation kit (Life Technologies) according to the manufacturer's protocol. For unstimulated versus stimulated comparisons, primary T lymphocytes were stimulated in anti-CD3-coated flasks (20 μg ml^−1^ OKT3, sourced in-house) with 1 μg ml^−1^ anti-CD28 (CD28.2, BioLegend) and 50 ng ml^−1^ IL-2 (AbD Serotec). For methyl-SILAC labelling, primary T lymphocytes were cultured with 2 μg ml^−1^ phytohemagglutinin (Sigma) and 50 ng ml^−1^ IL-2 (AbD Serotec). Primary T lymphocytes were cultured for 7–9 days at 37 °C in a humidified 5% CO_2_ atmosphere, medium was supplemented with 100 U penicillin and 100 μg ml^−1^ streptomycin (PAA). Methyl-SILAC-labelled cells were mixed 1:1 before collecting. Cells were collected, washed with phosphate-buffered saline and snap-frozen.

### Enrichment of arginine-methylated peptides

100 × 10^6^ Jurkat cells or 200 × 10^6^ primary T lymphocytes were lysed in 500 μl 8 M urea in 20 mM Tris pH8 at room temperature (RT) for 15 min. Lysates were sonicated four times for 10 s with a micro-tip sonicator set at 10 W and centrifuged at 20,000 *g* for 15 min at RT. The supernatant was collected and proteins reduced with 5 mM DTT for 30 min, then alkylated with 15 mM chloroacetamide for 30 min at RT. Protein was quantified using the Bradford dye binding assay. In the unstimulated–stimulated comparison, unstimulated T cells from three donors were pooled and lysed for each biological replicate, giving a total of 400 × 10^6^ unstimulated cells per replicate. An equal number of stimulated cells from the same donors were lysed and protein from unstimulated cells was mixed with stimulated cells 1:1. A total of nine donors were used, divided into three biological replicates. Urea was diluted to 2 M (trypsin digests) or 1 M (chymotrypsin, GluC digests). CaCl_2_ (1 mM) was added to the tryptic digests. Protease:protein ratios were in the range 1:100–1:50. Proteins were digested overnight at 37 °C. Digests were acidified to 0.1% TFA and centrifuged at 2,000 g for 5 min. Each 10 mg of peptides was desalted using 500 mg Sep-Pak C18 cartridges (Waters), eluting with 40% acetonitrile 0.1% TFA. Peptides were lyophilized and each 10 mg was resuspended in 1.3 ml immuno-affinity purification (IAP) buffer (50 mM MOPS, 10 mM Na_3_PO_4_, 50 mM NaCl, pH 7.2). For each IAP, 200 μg D5A12 antibody (CST # 8015, Cell Signaling Technology, Danvers, MA) or 270 μg MeR4100 antibody (CST # 8711, Cell Signaling Technology, Danvers, MA) was bound to 80 μl protein A agarose beads (Roche) for 3 h at RT. For unstimulated–stimulated IAPs and IAPs before strong cation exchange (SCX), 100 μg D5A12 and 135 μg MeR4100 were used in combination. Resuspended peptides were centrifuged at 20,000 *g* for 5 min, added to the beads and rotated overnight at 4 °C. Beads were washed three times with 1 ml IAP buffer and once with water. Peptides were eluted with 2 × 0.15% TFA and passed through a 1.6 μm pore-size glass microfibre plug. For SCX fractionation, peptides were dried and resuspended in 10 mM KH_2_PO_4_, 20% acetonitrile, pH 2.7. SCX was performed in tips packed with SCX phase (Empore) using elutions of 30 mM, 50 mM, 70 mM, 100 mM, 160 mM, 350 mM, 500 mM KCl in 10 mM KH_2_PO_4_ 20% acetonitrile pH 2.7. Peptides were desalted with C18 (Empore)-packed tips and dried.

### HILIC fractionation

Supernatant remaining after immuno-affinity purification of arginine-methylated peptides from unstimulated–stimulated cells was desalted using 500 mg Sep-Pak C18 cartridges (Waters). Peptides were dried, resuspended in 85% acetonitrile, 0.1% formic acid and applied to a HILIC column (ZIC HILIC 150 × 4.6 mm, 3.5 mm, 200 Å, Merck). Fractionation was performed at 0.5 ml min^−1^ with buffer A (0.1% formic acid) and buffer B (95% acetonitrile, 0.1% formic acid) as follows: 0–4.5 column volumes 90% buffer B, 4.5–14.5 column volumes gradient to 40% buffer B, 14.5–17 column volumes 40% buffer B, 17–17.1 column volumes 90% buffer B, 17.1–20 column volumes 90% buffer B.

### Mass spectrometry data acquisition

Peptides were resuspended in 0.1% TFA and analysed on an Ultimate RSLC nano (Dionex) system run in line with a Q Exactive mass spectrometer (Thermo Scientific). Peptides were resolved on a C18 reverse phase 50 cm × 75 μm Easy-Column (Thermo Scientific) using a linear gradient of 5–44% Buffer B (80% acetonitrile, 0.1% formic acid) at 300 nl min^−1^ over 130 min. The mass spectrometer was operated in a ‘Top 10' data-dependent acquisition mode with dynamic exclusion enabled (40 s). Survey scans (mass range 300–1,650 Th) were acquired at a resolution of 70,000 at 200 Th with the 10 most abundant multiply charged (*z*≥2) ions selected with a 3-Th isolation window for HCD fragmentation. MS/MS scans were acquired at a resolution of 17,500 at 200 Th.

### Mass spectrometry data analysis

MS/MS spectra were de-isotoped and charge deconvoluted with Progenesis ClearSpec (Nonlinear Dynamics). The .mgf files were uploaded to the UTSouthwestern Central Proteomics Facility Pipeline^40^ and searched against a concatenated and reversed decoy UniProt human database with OMSSA, X! Tandem native scoring and X! Tandem kscore. Cysteine carbamidomethylation and ^13^C_4_-labelled methionine or methyl-^13^CD_3_-labelled methionine were set as fixed modifications. Acetylation of protein amino (N)-termini, methionine oxidation, light monomethylarginine, light dimethylarginine, heavy monomethylarginine, heavy dimethylarginine were set as variable modifications. Precursor mass tolerance was 20 p.p.m., fragment mass tolerance was 0.1 Da. Localization of arginine methylation was performed using ModLS^41^. A maximum of three mis-cleavages were allowed for tryptic specificity, two mis-cleavages were allowed for chymotrypsin and GluC. FDR was set to 5% at the peptide level and results filtered for arginine-methylated peptides. Raw data files were searched for matching methyl SILAC pairs using a programme developed in-house (MethylQuant, programme available on request). Peptides were retained if they satisfied the following criteria: a matching methyl SILAC pair at a 1:1 intensity ratio, mass error <9.3 p.p.m., IP probability score >0.25 and ModLS score >8. When the ModLS score was <8, the peptide was retained if: all arginines were methylated, all methylated arginines were mis-cleaved (tryptic digests) or all methylated arginines had a PTMscore ≥0.95. After these filters, the FDR was calculated by determining the proportion of decoy methylated peptide spectrum matches that matched to a methyl-SILAC pair. FDR for arginine-methylated peptides was 0.93%. To generate Fig. 1c, identified methylated peptides, including decoy hits for calculation of FDR, generated from experiments on heavy methyl-SILAC-labelled primary T cells and iMethyl-SILAC-labelled primary T cells were sequentially filtered at a range (0.01 to 1.0, interval=0.01) of iProphet probability thresholds (a measure of identification confidence). For the subset of retained peptides at each threshold, the FDR was calculated. All raw data files, fragmentation spectra and identified peptides can be accessed at ftp://PASS00593:CM5498jzp@ftp.peptideatlas.org/. Unstimulated–stimulated data were analysed with MaxQuant 1.4.1.2 (ref. 42), Lys8 and Arg10 were set as labels, re-quantify was enabled. Cysteine carbamidomethylation was set as a fixed modification, acetylation of protein N-termini and methionine oxidation were set as variable modifications. Monomethylarginine and dimethylarginine were set as additional variable modifications for methylarginine IAPs. Enzyme specificity was set to Trypsin/P with three mis-cleavages allowed. First search was at 20 p.p.m., main search at 4.5 p.p.m., PSM FDR was set to 1%. Evidence.txt file was filtered to retain methylated peptides with scores >62, PTM localizations >0.75 and those which had been previously identified in methyl-SILAC experiments. Median extracted ion currents (XICs) for light and heavy modified peptides were calculated within each biological replicate, quantification was required in at least two of three biological replicates. Changes in methylation occupancy were calculated by dividing the heavy intensity for a methylated peptide with the normalized Heavy/Light (H/L) ratio of the corresponding protein. Differentially methylated peptides and differential occupancy were determined using the LIMMA package with Loess and quantile normalization^43^. A threshold *P* value of 0.01 was used, adjusted for multiple testing with the Benjamani–Hochberg method^44^. Sequence motifs were generated using pLOGO^23^, various significantly overrepresented amino acids were fixed to generate motif subsets. Overrepresented biological processes were calculated using ProteinCenter (Thermo Scientific).

## Author contributions

V.G. performed all the experiments and conceived the isomethionine labelling strategy. V.G. and O.A. designed the experiments. V.G., D.T. and O.A. analysed the data. V.G. wrote MethylQuant. A.G. provided the antibodies and expertise with immuno-affinity purifications. B.T. provided expertise with sample preparation and data analysis. V.G. and O.A. wrote the manuscript.

## Additional information

**How to cite this article:** Geoghegan, V. *et al.* Comprehensive identification of arginine methylation in primary T cells reveals regulatory roles in cell signalling. *Nat. Commun.* 6:6758 doi: 10.1038/ncomms7758 (2015).

## Supplementary Material

Supplementary Figures Supplementary Figures 1-4

Supplementary Data 1 All unique identified methylated peptides from Jurkat or primary human T cells matched to a methlyl SILAC pair

Supplementary Data 2 All unique methylated proteins identified together with the methylation sites

Supplementary Data 3 Transcription factors with a role in T cell differentiation found to be methylated on arginine

Supplementary Data 4 Changes in arginine methylation during primary human T cell stimulation and differentiation, before normalising for changes in protein levels

Supplementary Data 5 Changes in arginine methylation during primary human T cell stimulation and differentiation, after normalising for changes in protein levels (change in methylation occupancy)
