# Supplementary material for: Comprehensive identification of arginine methylation in primary T cells reveals regulatory roles in cell signalling
Source: Nat Commun. 2015 Apr 7;6:6758. doi: 10.1038/ncomms7758 (PMC4396391; doi:10.1038/ncomms7758)
Supplement: Supplementary Figures — 1-4 [file ncomms7758-s1.pdf]

Supplementary Figure 1

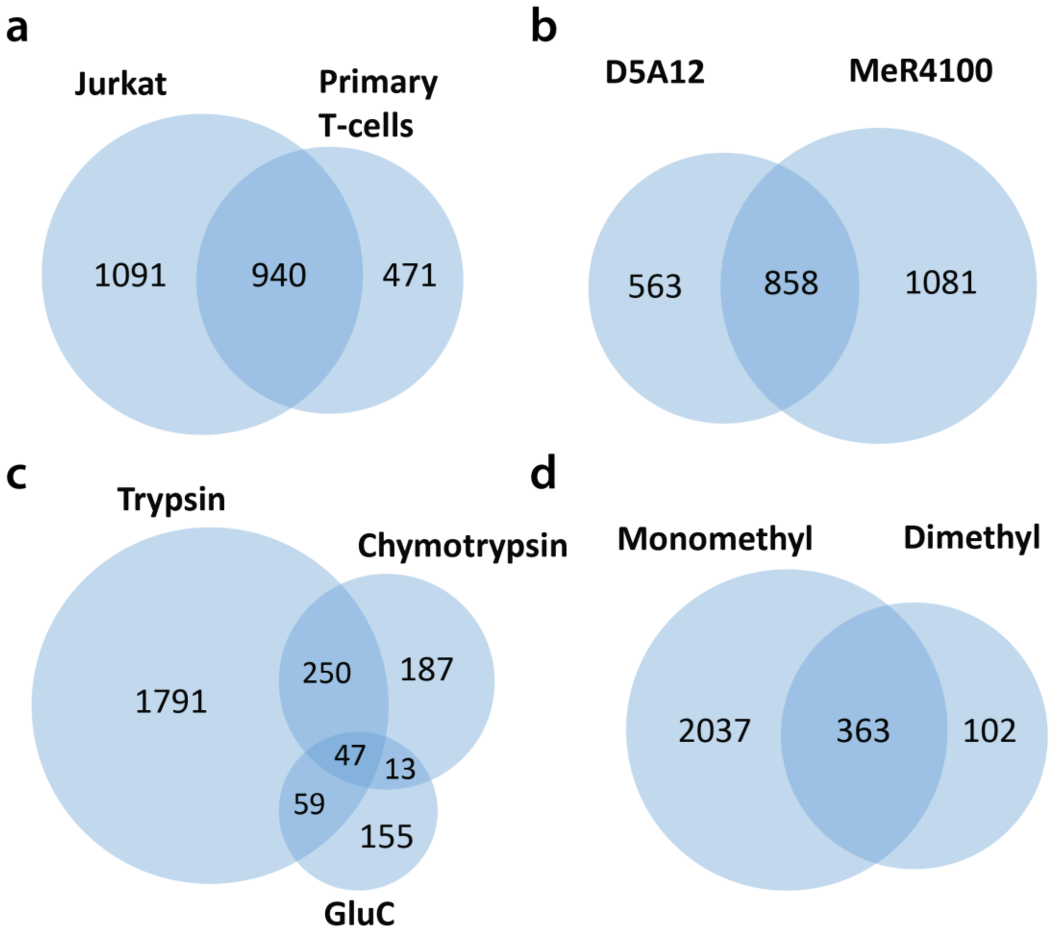

Supplementary Figure 1: Breakdown of all identified arginine methylation sites. (a) Overlap between identified arginine methylation sites in Jurkat T cells and primary T cells. (b) Arginine methylation sites identified by each antibody. (c) Arginine methylation sites identified after digestion with trypsin, chymotrypsin or GluC. (d) Type of arginine methylation site identified.

Supplementary Figure 2

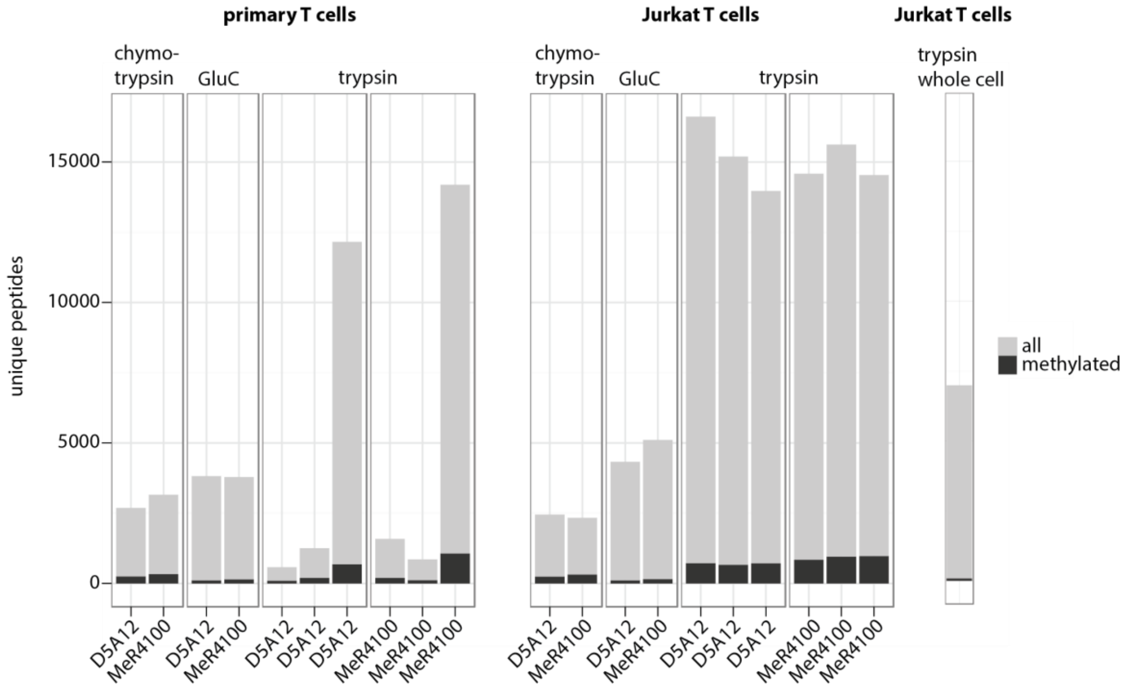

Supplementary Figure 2: Unique peptides identified across all immuno-affinity purifications, grouped by protease (chymotrypsin, GluC or trypsin) and source material (Jurkat T cells or primary T cells). For comparison, results from a representative iMethyl-SILAC labelled whole cell lysate are shown.

Supplementary Figure 3

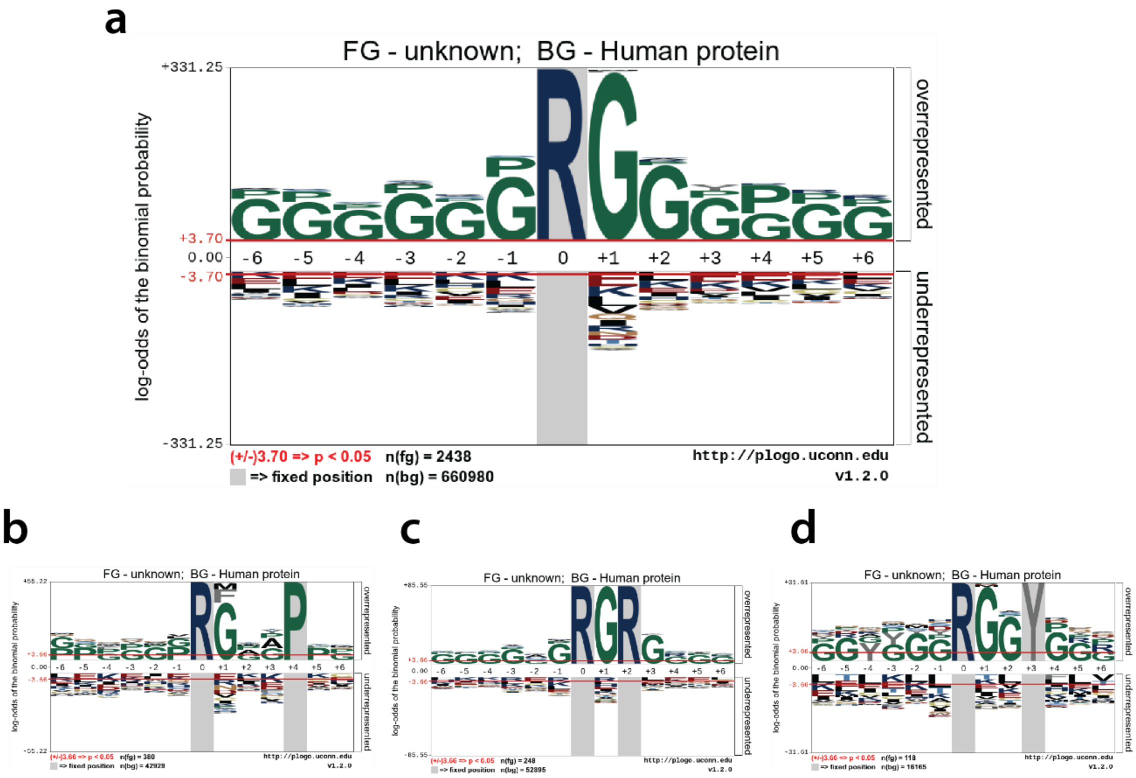

Supplementary Figure 3: Arginine methylation sequence context. All methylation sites were analysed with pLOGO to generate a motif. Significantly over represented amino acids were fixed to explore motif sub-sets. (a) Sequence motif generated from all arginine methylation sites. (b) Proline at +4 was fixed to explore the (P)Rxxx(P) motif. (c) Arginine at +2 was fixed to explore the RxR motif. (d) Tyrosine at +3 was fixed.

Supplementary Figure 4

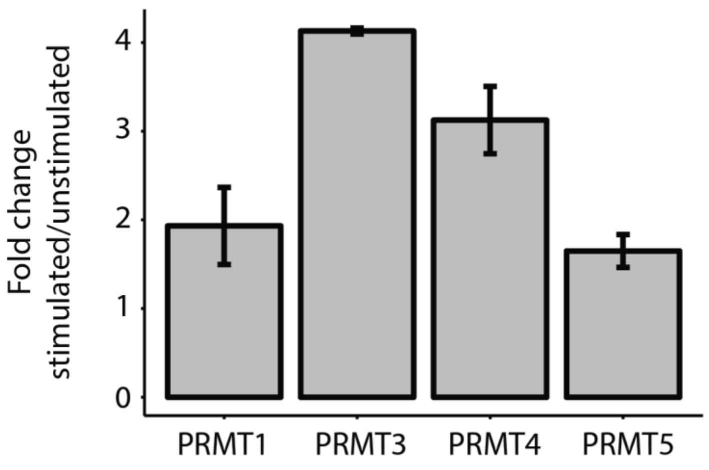

Supplementary Figure 4: PRMTs quantified during T-lymphocyte stimulation. Fold changes (stimulated/unstimulated) measured by mass spectrometry were averaged across all biological replicates. Bars are ±1SD.
